# Full retraction: Sasirekha Krishnan, Shruthi Lakshmanan, Geetha Krishnan Iyer, Krishnan UmaMaheswari, Subramanian Krishnakumar. The role of signaling pathways in the expansion of corneal epithelial cells in serum-free B27 supplemented medium. Mol Vis. 2010; 16:1169-1177.

**Published:** 2020-08-18

Data presented in Figure 4 was misrepresented. This discrepancy was brought to our attention by the corresponding author.

On this basis, the Editors formally retract this article from Molecular Vision.

Sincerely,

The Editors of Molecular Vision

